# Direct retrieval of isoprene from satellite-based infrared measurements

**DOI:** 10.1038/s41467-019-11835-0

**Published:** 2019-08-23

**Authors:** Dejian Fu, Dylan B. Millet, Kelley C. Wells, Vivienne H. Payne, Shanshan Yu, Alex Guenther, Annmarie Eldering

**Affiliations:** 1 Jet Propulsion Laboratory, California Institute of Technology, Pasadena, CA 91109 USA; 2 University of Minnesota, St. Paul, MN 55108 USA; 3 University of California, Irvine, CA 55108 USA

**Keywords:** Atmospheric science, Atmospheric chemistry

## Abstract

Isoprene is the atmosphere’s most important non-methane organic compound, with key impacts on atmospheric oxidation, ozone, and organic aerosols. In-situ isoprene measurements are sparse, and satellite-based constraints have employed an indirect approach using its oxidation product formaldehyde, which is affected by non-isoprene sources plus uncertainty and spatial smearing in the isoprene-formaldehyde relationship. Direct global isoprene measurements are therefore needed to better understand its sources, sinks, and atmospheric impacts. Here we show that the isoprene spectral signatures are detectable from space using the satellite-borne Cross-track Infrared Sounder (CrIS), develop a full-physics retrieval methodology for quantifying isoprene abundances from these spectral features, and apply the algorithm to CrIS measurements over Amazonia. The results are consistent with model output and in-situ data, and establish the feasibility of direct global space-based isoprene measurements. Finally, we demonstrate the potential for combining space-based measurements of isoprene and formaldehyde to constrain atmospheric oxidation over isoprene source regions.

## Abstract (web summary)

Isoprene is a key component of the atmosphere, with impacts on oxidation, ozone and organic aerosols, but in-situ measurements are limited. Here, the authors present a full-physics measurement framework based on satellite data that enables the direct observation of atmospheric isoprene from space.

## Introduction

Isoprene (C_5_H_8_) is the predominant nonmethane volatile organic compound (VOC) emitted to the atmosphere, with an estimated global flux of ~500^1^ Tg year^−1^, several-fold greater than all anthropogenic VOCs combined^2^. Once emitted, isoprene plays a major role in ozone formation^3^, organic aerosol production^4^, and the cycling of reactive nitrogen^5^. Isoprene is also a hydroxyl radical (OH) sink, and alters the atmosphere’s oxidizing capacity in a way that depends intricately on NO_*x*_^6,7^.

Present scientific understanding of isoprene emissions and their impacts on tropospheric composition is limited in several fundamental respects. In situ isoprene measurements are sparse, particularly over the tropics, thought to be the predominant global source region. Bottom-up emission inventories are derived mainly from extrapolation of these limited point measurements, and carry large uncertainties—especially for tropical forests and other regions of high biodiversity. As a result, isoprene emission estimates vary substantially: Arneth et al.^8^ showed that global isoprene flux estimates can vary by nearly fivefold across current emission models and input datasets. Furthermore, the degree to which isoprene acts as a net OH sink, thus affecting atmospheric oxidation (and its own lifetime) is not yet fully understood.

Formaldehyde (HCHO), as a high-yield isoprene oxidation product that is observable from space, has been widely used as a proxy for investigating isoprene emissions^9–11^. Global satellite-based formaldehyde observations have been available since 1996^12^, and are presently feasible using the Ozone Monitoring Instrument (OMI, 2004–), Global Ozone Monitoring Experiment-2 (GOME-2A, B, C 2006–), Ozone Mapping Profiler Suite (OMPS, 2011–), and TROPOspheric Monitoring Instrument (TROPOMI, 2017–), providing spatial and temporal coverage beyond what is achievable with in situ measurements. However, formaldehyde is also produced from other VOCs, and thus is not a unique marker for isoprene. This presents particular challenges over fire regions, and over populated areas with their substantial anthropogenic VOC sources. The use of formaldehyde as an isoprene proxy is also hindered by incomplete knowledge of the non-linear chemistry linking isoprene and OH^13^, and of the NO_*x*_-dependence governing the formaldehyde yield and its production timescale^14^.

Recent laboratory measurements of the isoprene cross-section in the thermal infrared (IR)^15^ have provided the spectroscopic parameters needed for the remote sensing of isoprene from space. Here, we apply these spectroscopic data and demonstrate the detection of the isoprene spectral signature in space-borne radiance measurements from the Cross-track Infrared Sounder (CrIS), an imaging Fourier transform spectrometer onboard the Suomi National Polar-Orbiting Partnership (NPP) satellite. We present a full-physics algorithm for retrieving atmospheric isoprene columns from the CrIS data, and use the two-step retrieval strategy to quantify the isoprene distribution over Amazonia, a major source region. Finally, we discuss the information content and uncertainty characteristics for these new isoprene measurements, and compare the satellite observations with in situ data and model predictions for the same region.

## Results

### CrIS description

CrIS covers three IR spectral regions spanning 650–2550 cm^−1^ (1.25 cm^−^^1^ spectral resolution), in each case with a 3 × 3 array of circular sensing apertures in the focal plane forming nine fields of view having 14 km diameter at nadir (41 × 24 km at the scan extremes). The CrIS instrument was launched on-board the Suomi-NPP satellite into a sun-synchronous orbit (daytime overpass at 13:30 local time) on October 28, 2011, and conducts cross-track scanning with 2200 km swath width, providing near-global coverage twice daily. The CrIS Level 1B data products, including single-footprint calibrated radiance spectra, geolocation information, metadata, and a suite of derived parameters related to the observations, have been operationally produced, validated, and publicly released by both NASA and NOAA^16–20^. The highly accurate spectral and radiometric performance^16–19^, low-instrument noise^20^, afternoon overpass, and consistency with other IR sounders^19^ make CrIS a powerful instrument for remote sensing of atmospheric composition^21,22^ and for isoprene in particular—despite the fact that it was developed primarily to support numerical weather predictions via improved temperature and water vapor profile retrievals.

Space-based measurement of atmospheric isoprene is made challenging by its weak absorption (~0.02–0.15 K spectral signal level) and by interferences from other species active in the same spectral range. Our analysis focuses on the ν_27_ and ν_28_ isoprene bands (860–940 cm^−1^), since these are the strongest absorption features, and subject to the least influence from interfering atmospheric species, among its 33 reported IR spectral bands^15^ between 600 and 6500 cm^−1^.

### Isoprene spectral signal and role of interferences

**Fig. 1:**
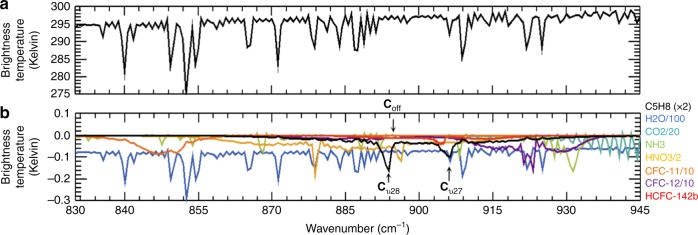
Example CrIS spectrum and relevant speciated spectral features. **a** An individual CrIS-measured spectrum (black line) in the region of isoprene’s ν_27_ and ν_28_ bands, obtained over Amazonia (8.698°S, 69.134°W) on September 30, 2014. The major features are associated with H_2_O vapor. **b** Spectral contributions from isoprene (C_5_H_8_), water vapor (H_2_O), carbon dioxide (CO_2_), nitric acid (HNO_3_), ammonia (NH_3_), and chlorofluorocarbons (CFC-11 and CFC-12) for the same scene. **C**_ν28_: spectral channel near the peak of isoprene absorption for the ν_28_ band. **C**_off_: closest spectral channel to **C**_ν28_ that is near the continuum featuring no isoprene absorption. Spectral features for H_2_O, CO_2_, HNO_3_, CFC-11, and CFC-12 have been divided by 100, 20, 2, 10, and 10, respectively

Figure 1 shows an example individual spectrum measured by CrIS over Amazonia (8.698°S, 69.134°W; 09/30/2014). Also shown are the spectral contributions from relevant absorbers in the vicinity of the isoprene ν_27_ and ν_28_ bands for the same scene. The latter are computed using the radiative transfer model (RTM, see Methods) implemented in the MUlti-SpEctra, MUlti-SpEcies, MUlti-SEnsors (MUSES, see Methods)^21–24^ full-physics retrieval algorithm—enabling simulation of the trace gas radiances based on the viewing geometry and spectral sampling grid of the CrIS instrument. The Methods section provides relevant details on the configuration of the RTM used for simulating the CrIS observations. We see that, along with water vapor, HNO_3_, NH_3_, and CFC-12 all have absorption features near those of isoprene. Accurate treatment of these interferences is thus crucial for deriving isoprene abundances from the IR spectra.

We also investigated the potential for other VOCs to interfere with the isoprene signal used here. Compounds that are also emitted from vegetation, that contain carbon double bond structures analogous to those causing isoprene’s ν_27_ and ν_28_ features (terminal = CH_2_ moieties), and that have published absorption cross-sections in the relevant spectral region, include ethene, β-pinene, d-limonene, and myrcene. Supplementary Fig. 1 compares the cross-sections for the latter three, plus α-pinene, to that of isoprene (for ethene see Coheur et al.^25^). We find that the resulting spectral signals within 830–945 cm^−1^ are minor (<0.01 K) for all of these compounds due to their much weaker (~4–100×) absorption cross-sections compared to isoprene, plus their lower expected concentrations over our region of study (Supplementary Note 1).

We examine the isoprene spectral signal relative to other absorbers in the native CrIS radiances (L1B product version 1.0, see Data Availability section) using a simple, fast, brightness temperature difference (Δ**T**_b_) metric. Analogous metrics have been used by the Infrared Atmospheric Sounder Interferometer (IASI) community for obtaining global maps of sulfur dioxide^26^, ammonia^27^, and methanol^28^. Here, we employ global cloud-screened CrIS data from September 2014, and compute Δ**T**_b_ as the difference in brightness temperature between the peak of absorption (**C**_ν28_ spectral channel; Fig. 1b) for the isoprene ν_28_ band and proximate spectral channels near the continuum (**C**_off_; Fig. 1b) where no isoprene absorption is found. Cloud screening was performed using the CrIS 900 cm^−1^ brightness temperature (**T**_b,900_), surface skin temperature (**T**_skin_), and water vapor column abundance (**Ω**_H2O_), with **T**_skin_ and **Ω**_H2O_ taken from Modern-Era Retrospective analysis for Research and Applications-2 (MERRA-2) reanalysis^29^ and linearly interpolated to the CrIS overpass time. When the **T**_b,900_ − **T**_skin_ difference at a given **Ω**_H2O_ is smaller than the corresponding threshold value, the observation is flagged as cloudy and excluded from further analysis. Thresholds were defined based on the simulated relationship between **T**_b,900_ − **T**_skin_ and **Ω**_H2O_ under clear-sky conditions.

**Fig. 2:**
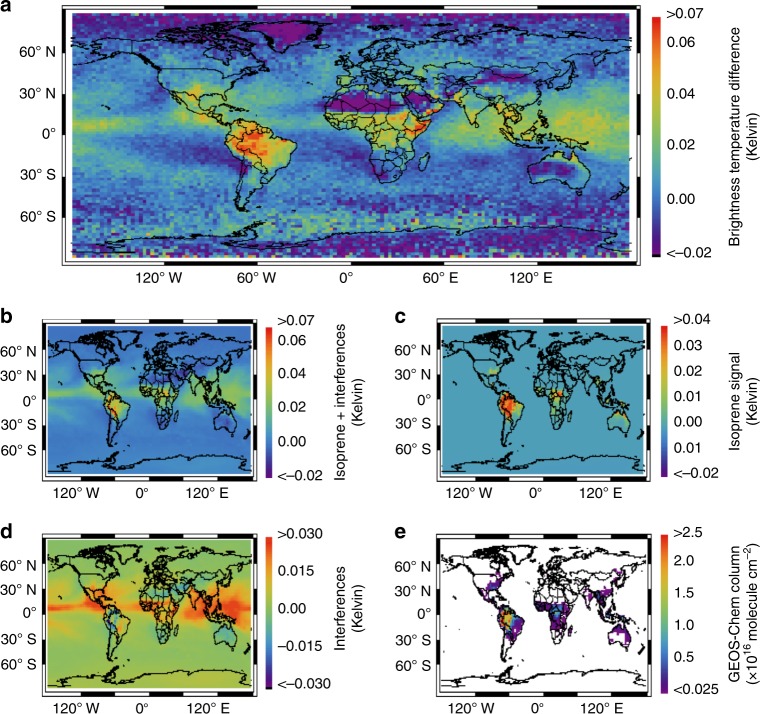
Accounting for spectral interferences in the quantification of isoprene. **a** CrIS brightness temperature differences (Δ**T**_b_) for September 2014 (daytime, cloud-screened data only) mapped on a 2.0° × 2.5° latitude–longitude grid. Δ**T**_b_ values are calculated as the spectral difference between the peak of absorption and proximate near-continuum data for the isoprene ν_28_ band. **b** Simulated Δ**T**_b_ distribution predicted based on GEOS-Chem model isoprene columns for the same month. **c** Simulated brightness temperature differences at the peak of the ν_28_ isoprene absorption (Δ**T**_ν28_) between an isoprene-free atmosphere and an atmosphere containing the GEOS-Chem isoprene distribution, based on an observing system simulation experiment. Panel **c** represents the spectral signal in the CrIS data arising purely from isoprene that would be expected given the GEOS-Chem isoprene distribution. **d** Difference between panels (**b**) and (**c**). Panel **d** represents the contributions from interfering species to Δ**T**_b_ when calculated from the on-peak (ν_28_) vs. off-peak radiances as in panels (**a**) and (**b**). **e** Isoprene column densities (10^16^ molecule cm^−2^) predicted by GEOS-Chem for the same month and spatial grid

Figure 2a maps the resulting CrIS Δ**T**_b_ data for September 2014 on a global 2.0° × 2.5° latitude–longitude grid. Only daytime overpass data are included due to their generally positive thermal contrast and thus greater sensitivity in the lower troposphere where most isoprene resides. We see in the Figure enhanced Δ**T**_b_ values above areas predicted by the GEOS-Chem chemical transport model (CTM)^30–32^ (Fig. 2e; see Methods) to have the largest isoprene column abundances (e.g., Amazon forest, central Africa, and Southeast Asia), reflecting the influence of the isoprene spectral signature in the CrIS Δ**T**_b_ data. However, Fig. 2a also shows Δ**T**_b_ enhancements over regions that are not predicted to have any appreciable isoprene (e.g., tropical oceans and Horn of Africa). Over tropical oceans, the predominant nonisoprene signal is due to water vapor and residual clouds. Spectrally varying surface emissivity can also influence the Δ**T**_b_ signals, and this effect is apparent in Fig. 2a over certain subtropical and arid regions (e.g., Yemen, Ethiopia, and Somalia). However, it should also be noted that the model isoprene estimates over many of these regions are highly uncertain, since there is often (e.g., for East Africa) a complete lack of in situ emission measurements for the regionally dominant plant species. This fact highlights the potential of the CrIS measurements for improving current models of atmospheric composition.

We conducted an observing system simulation experiment to more quantitatively interpret the above results, and to assess the degree to which meaningful isoprene information can be extracted from the CrIS spectra. To this end, we built new functionalities into the MUSES algorithm (see Methods) to allow computation of the Δ**T**_b_ distribution that would be expected given the isoprene abundances predicted by GEOS-Chem for September 2014. The resulting simulated brightness temperature differences (Fig. 2b) show strong spatial consistency with the observed values (Fig. 2a; satellite:model Pearson’s *r* = 0.68)_._ In particular, we see similar simulated and observed enhancements over high-isoprene regions such as Amazonia, as well as over low-isoprene regions such as the tropical Pacific. This coherence shows that the main factors driving the CrIS radiances in this part of the IR spectrum are well-represented in the RTM, while also emphasizing the combined importance of isoprene and of other atmospheric and surface properties in determining the Δ**T**_b_ values when calculated in this way.

The expected spectral signal arising specifically from isoprene is shown in Fig. 2c, which maps the simulated difference in brightness temperature at the peak of the ν_28_ isoprene absorption (Δ**T**_ν28_) between an isoprene-free atmosphere and an atmosphere containing the GEOS-Chem isoprene distribution. Figure 2d shows the simulated Δ**T**_ν28_ − Δ**T**_b_ difference, illustrating the corresponding nonisoprene contributions. Together, Fig. 2c, d show that isoprene signals can be reliably extracted from space-based observations at signal levels up to ~0.1 K, provided that the spectral influences from water vapor and other relevant factors are reliably accounted for. In the following section we describe a full-physics algorithm developed expressly for this purpose, and apply it to retrieve isoprene column abundances over Amazonia, which is the strongest isoprene source region apparent from the data in Fig. 2.

### CrIS isoprene retrievals and evaluation

We employ the MUSES algorithm to retrieve isoprene abundances directly from the thermal IR radiances measured by the CrIS satellite sensor. MUSES is a full-physics retrieval algorithm following optimal estimation principles^33^. It minimizes the differences between observed and measured radiances, subject to a priori knowledge, to infer the optimal or maximum a posteriori state vector ${\hat{\mathbf{x}}}$—which represents the atmospheric concentration of isoprene and of other absorbers within the spectral region of interest, along with relevant surface and cloud properties. The “Methods” section provides details on the algorithm’s theoretical basis and key equations.

The retrieval consists of two steps. The first step quantifies the spectral interferences arising from nonisoprene species (H_2_O, HNO_3_, and NH_3_) and from the Earth surface and clouds. This step employs spectral regions suitable for quantifying the relevant interferences, but that contain negligible isoprene absorption. The second step retrieves the isoprene abundances, and employs a spectral region containing the full ν_27_ and ν_28_ bands (see Table 1).

**Table 1:** Spectral regions used in the pre-isoprene and isoprene retrieval steps

Retrieval step^a^	Spectral data	Frequency		Spectral resolution	Spectral contributors
		Start (cm^−^^1^)	End (cm^−1^)		
Pre-isoprene step	CrIS level 1B data^b^	862.500	890.625	1.25 cm^−^^1^	H_2_O, HNO_3_, NH_3_, CO_2_, CFC-11, CFC-12, HCFC-142b^c^, surface emissivity, surface temperature, cloud optical depth, cloud height
		895.625	903.750		
		907.500	940.000		
Isoprene step		862.500	940.000		Isoprene, and all species in the pre-isoprene step

^a^The pre-isoprene retrieval step quantifies the spectral interferences arising from other species. The subsequent retrieval step quantifies the isoprene abundance

^b^CrIS single-footprint infrared geolocated and calibrated radiance data are used directly rather than level 2 cloud-cleared spectra, which are calculated using nine adjacent CrIS infrared footprints. Using single-footprint spectra improves the horizontal resolution of the CrIS retrievals from ~45 to ~13.5 km at nadir, thus enhancing representation of horizontal details

^c^HCFC-142b was not included in this analysis as its impact is minor for our study domain and timeframe, but due to its increasing trend it should be accounted for in future operational data production

**Fig. 3:**
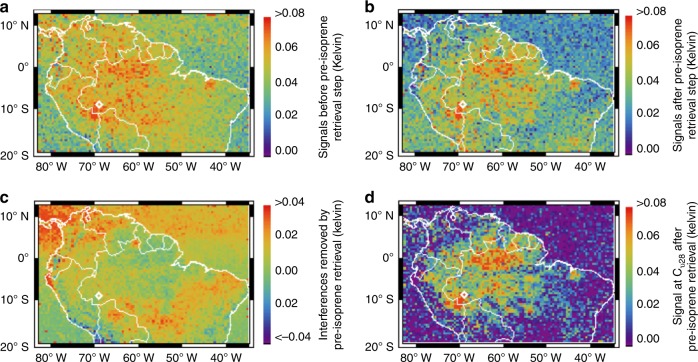
Isoprene spectral signal in CrIS measurements over Amazonia during September 2014, mapped on a 0.5° × 0.5° latitude–longitude grid. **a** Measured on-peak vs. off-peak brightness temperature differences (Δ**T**_b_) between the **C**_off_ and **C**_ν28_ spectral channels before the pre-isoprene retrieval step. **b** Measured Δ**T**_b_ between the **C**_off_ and **C**_ν28_ spectral channels after the pre-isoprene retrieval step. **c** Difference between panels **a** and **b** illustrating the interferences that are accounted for by the preisoprene retrieval step. **d** Brightness temperature difference Δ**T**_ν28_ at the **C**_ν28_ spectral channel between the CrIS-measured radiances and the model-simulated radiances from the pre-isoprene retrieval step, illustrating the spectral signal due to isoprene

We focus our analysis over Amazonia during September 2014 (the dry season). Figure 3a shows the native CrIS-measured Δ**T**_b_ over this region (representing the on-peak vs. off-peak radiance difference at the ν_28_ isoprene feature). As in Fig. 1a, spectral enhancements associated with isoprene (e.g., over northern and western Brazil) and with nonisoprene interferences (e.g., over tropical oceans) are clearly visible. Figure 3b shows the CrIS Δ**T**_b_ distribution after completion of the preisoprene retrieval step. As we see, after relevant nonisoprene spectral influences have been properly accounted for (these are shown in Fig. 3c), the isoprene signal clearly emerges in the CrIS radiances.

Figure 3d shows the brightness temperature difference computed at the peak of the ν_28_ absorption band (Δ**T**_ν28_) between the CrIS-observed radiances and the RTM-simulated radiances after the pre-isoprene retrieval step. Again, we see the isoprene signal clearly emerging in the CrIS data. Importantly, the patterns shown in Fig. 3d (as well as 3b) reflect CrIS spectral data corrected for nonisoprene interferences—but without incorporating any a priori information related to isoprene itself. Furthermore, the enhanced signal-to-noise apparent in Fig. 3d compared to Fig. 3a, b illustrates the power and increased sensitivity of the full-physics retrieval over empirical approaches relying on off-peak/on-peak spectral indices.

**Fig. 4:**
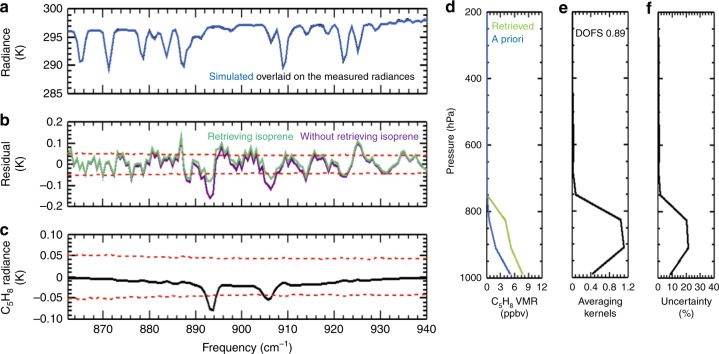
An example CrIS isoprene retrieval over Amazonia (8.698°S, 69.134°W) on September 30, 2014. **a** Calculated (blue) spectrum overlaid on the CrIS-measured (black) spectrum, plotted in brightness temperature units. The major features are due to H_2_O vapor. **b** Residual spectra (observed—calculated) after accounting for water vapor (H_2_O), nitric acid (HNO_3_), ammonia (NH_3_), and relevant surface and cloud properties. The purple line indicates the residual spectrum before accounting for isoprene (C_5_H_8_), clearly showing the isoprene signals peaking at 893.75 cm^−1^ (ν_28_ band) and 906.25 cm^−1^ (ν_27_ band). The green line depicts the residuals after fitting for isoprene. The red dashed lines denote the single-pixel CrIS noise level as reported in the CrIS L1B data product. **c** Difference between the residual spectra when including vs. excluding atmospheric isoprene in the fitting—illustrating the isoprene spectral signal in this example. **d** A priori (blue) and retrieved (green) isoprene volume mixing ratios (VMR). **e** Sum of the rows of the averaging kernel matrix for the isoprene retrieval, showing 0.9 degrees of freedom for signal (DOFS) in this example. **f** Estimated uncertainty of the retrieval

In the isoprene retrieval step, the MUSES algorithm uses the measured radiances from all spectral channels within the ν_27_ and ν_28_ bands to determine the isoprene abundance—thus fully maximizing the sensitivity and information content of the retrievals. Figure 4 presents an example of an individual CrIS retrieval over Amazonia (8.698°S, 69.134°W on 09/30/2014; location indicated by the white diamond in Fig. 3). The full-physics algorithm successfully minimizes the prominent spectral residuals due to isoprene (Fig. 4b, c), yielding the retrieved isoprene concentration shown in Fig. 4d. As part of the optimal estimation framework, the retrieval delivers key measurement characteristics for each observation, including averaging kernels (Fig. 4e) and uncertainties (Fig. 4f). Examination of the averaging kernel matrix for this example shows that the CrIS retrievals are primarily sensitive to isoprene in the 825–990 hPa pressure range. The degrees of freedom for signal (DOFS), corresponding to the trace of the averaging kernel matrix, is close to 1 for the example shown, demonstrating that the total isoprene column abundance can be determined from a single CrIS measurement over a strong source region such as Amazonia.

**Fig. 5:**
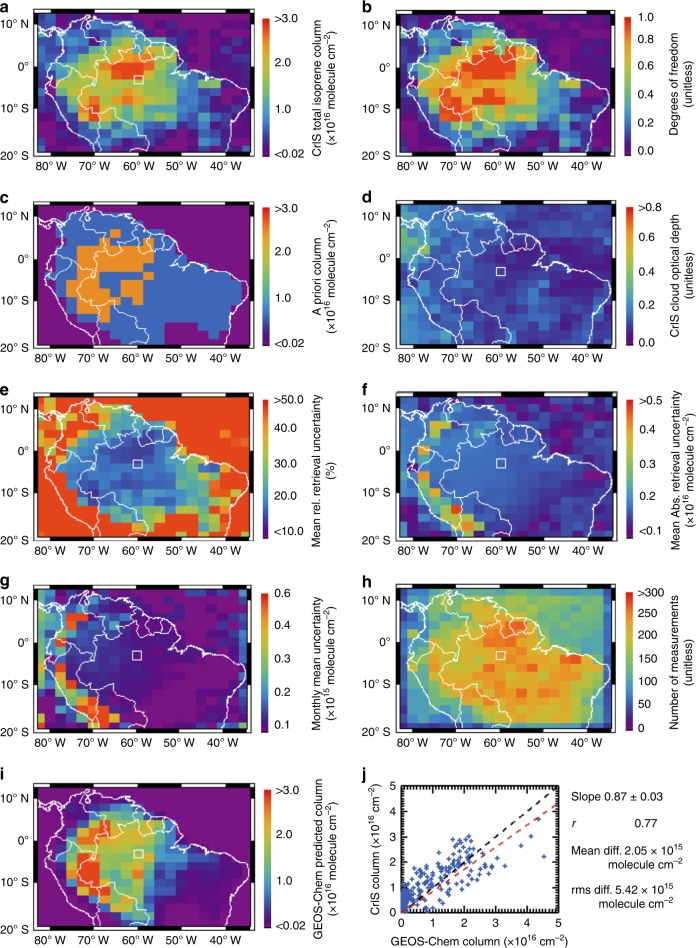
CrIS isoprene retrievals over Amazonia during September 2014. The white rectangle in panels a–i depicts the study domain for the airborne Green Ocean Amazon (GoAmazon) campaign during the same month. Data in panels **a**–**i** are mapped on a 2.0° × 2.5° latitude–longitude grid. **a** Isoprene column densities retrieved from CrIS. **b** Degrees of freedom for signal in the CrIS measurements. **c** A priori isoprene columns used in the retrievals. **d** Retrieved cloud optical depths. **e** Mean relative retrieval uncertainty (%). **f** Mean absolute retrieval uncertainty (10^16^ molecule cm^−2^). **g** Uncertainty in the monthly mean isoprene columns (retrieval uncertainty divided by $\sqrt n$; 10^15^ molecule cm^−2^). **h** Number of CrIS measurements per grid cell. **i** Isoprene column densities predicted by the GEOS-Chem model for the same period. **j** Correlation between the CrIS and GEOS-Chem isoprene column densities. The red dashed line shows the satellite:model linear fit, while the black dashed line indicates the 1:1 relationship

Figure 5a shows the monthly mean isoprene columns measured by CrIS over Amazonia during September 2014, based on ~70,000 single-footprint measurements averaged and mapped following standard procedures^22^. The observed spatial distribution of isoprene shows a broad enhancement over the Amazon basin, with peak concentrations to the north (~60–70°W, 0–5°S; corresponding to the Brazilian states of Amazonas, Pará, and Roraima) and west (~70°W, 10°S; states of Amazonas, Acre, and part of Peru). The DOFS range from approximately 0.5–1 over areas with elevated isoprene (Fig. 5b). The observed spatial distribution of isoprene diverges significantly from the a priori (Fig. 5c, Supplementary Fig. 2), reflecting the information content of the CrIS observations.

Clouds can represent a significant uncertainty source in satellite remote sensing of atmospheric composition^21–24^, and we thus plot in Fig. 5d the retrieved cloud optical depths for the same set of CrIS observations mapped in Fig. 5a. The CrIS data have been subjected to a two-stage cloud-clearing procedure, as follows. First, the fast **T**_b,900_ − **T**_skin_ screening described earlier is used as a first-order filter to remove cloudy scenes across the globe. Subsequently, in the pre-isoprene step, we explicitly retrieve relevant cloud parameters to account for residual thin clouds and to filter out scenes with cloud optical depth greater than one. With this treatment, cloud impacts over land are minor. Figure 5d shows that the resulting mean cloud optical depths are generally <0.2 over land, where their correlation with retrieved isoprene is negligible (*r* ~0.08). Some ocean regions (e.g., 0°–10°N, 78°W–80°W over the Pacific), however, feature cloud optical depths of up to ~0.6–0.7 that are associated with weak artificial enhancement in the retrieved isoprene columns. A more stringent cloud threshold would reduce such effects, and could be employed in postretrieval analysis.

Figure 5e, f map the total a posteriori retrieval uncertainty, including contributions from spectral noise, interferences, and smoothing errors. Values shown represent the mean across all CrIS measurements within a grid cell, and are dominated by smoothing errors—as expected given the DOFS and total column retrieval approach. The estimated uncertainty of a single-retrieval is generally ~0.25 × 10^16^ molecule cm^−2^ (Fig. 5f). The single-retrieval 3σ detection limit is ~0.8 × 10^16^ molecule cm^−2^. However, the random component of this uncertainty is strongly reduced by averaging given the dense spatial sampling provided by the CrIS instrument (Fig. 5h). We note that a posteriori uncertainties such as the above can underestimate the true measurement error, as they do not account for possible systematic errors in the retrieval framework (e.g., associated with the absorption cross-sections), and also depend on the prescribed a priori errors. As a result, retrieval evaluation against independent measurements is required. Later, we employ aircraft measurements obtained over our region of study for this purpose.

Simulated isoprene columns from the GEOS-Chem model (Fig. 5i) exhibit significant spatial coherence with the CrIS measurements and are of similar magnitude. The satellite:model correlation is *r* = 0.77, with a slope of 0.87 ± 0.03, mean difference of 2.05 × 10^15^ molecule cm^−2^, and root mean square (RMS) difference of 5.42 × 10^15^ molecule cm^−2^. Despite this broad-scale agreement, the CrIS measurements reveal a number of features that are not captured by the model. For instance, the large isoprene peak measured by CrIS over northern Brazil is missing from GEOS-Chem, which instead exhibits a peak farther to the north and west. Furthermore, the isoprene enhancement seen over western Brazil and eastern Peru is overestimated by the model. Finally, the CrIS data show that enhanced isoprene columns extend significantly farther south and east than is predicted. For example, total isoprene columns of ~1 × 10^16^ molecule cm^−2^ are observed over the region bounded by 10–20°S and 40–45°W. These are well above the estimated retrieval uncertainty, have DOFS (Fig. 5b) indicating meaningful sensitivity to atmospheric isoprene, and are nearly completely absent from the model. Thus, we anticipate that subsequent work applying these data can significantly advance the state of science related to isoprene emissions and their ensuing chemical impacts.

**Fig. 6:**
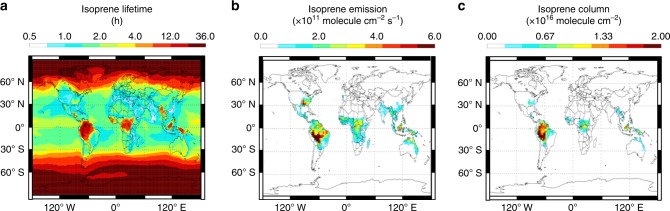
Global distribution of isoprene lifetime (**a**), emissions (**b**), and column abundance (**c**) as simulated by the GEOS-Chem model for September 2013. All data is mapped on a 2.5° × 2.0° latitude–longitude grid

The Green Ocean Amazon (GoAmazon) flight campaign (2014/2015) obtained in situ isoprene mixing ratio profiles via proton transfer reaction-mass spectrometry (PTR-MS) onboard the Gulfstream-1 (G-1) research aircraft^34,35^. During September 2014, airborne PTR-MS measurements were conducted over 15 days near and around Manaus, Brazil in central Amazonia (2.0–4.0°S, 59.0–61.0°W). Flights consisted of sequential level legs carried out perpendicular to the prevailing wind and at altitudes ranging from 500 to a maximum of 7100 m^34,35^. In order to evaluate the performance of the CrIS isoprene retrievals against these in situ measurements, we first selected those spatially and temporally coincident satellite–aircraft pairs having geolocation within a common 0.2° × 0.2° latitude–longitude grid cell and maximum time separation of 6 h. We note that the isoprene lifetime over Amazonia is uncertain, but based on the GEOS-Chem model could exceed 12 h (Fig. 6a) due to suppressed OH under low-NO_*x*_ and high-isoprene emissions.

The CrIS-retrieved isoprene profiles were then averaged over the 0–2 km altitude range, corresponding to the region of peak satellite sensitivity (Fig. 4e), and mapped onto the 0.2° × 0.2° grid. The airborne PTR-MS isoprene measurements, which show the majority (>95%) of the isoprene burden residing in the lowest 2 km (Supplementary Figs. 3–5), were likewise averaged and mapped via the same methodology used for the CrIS data.

**Fig. 7:**
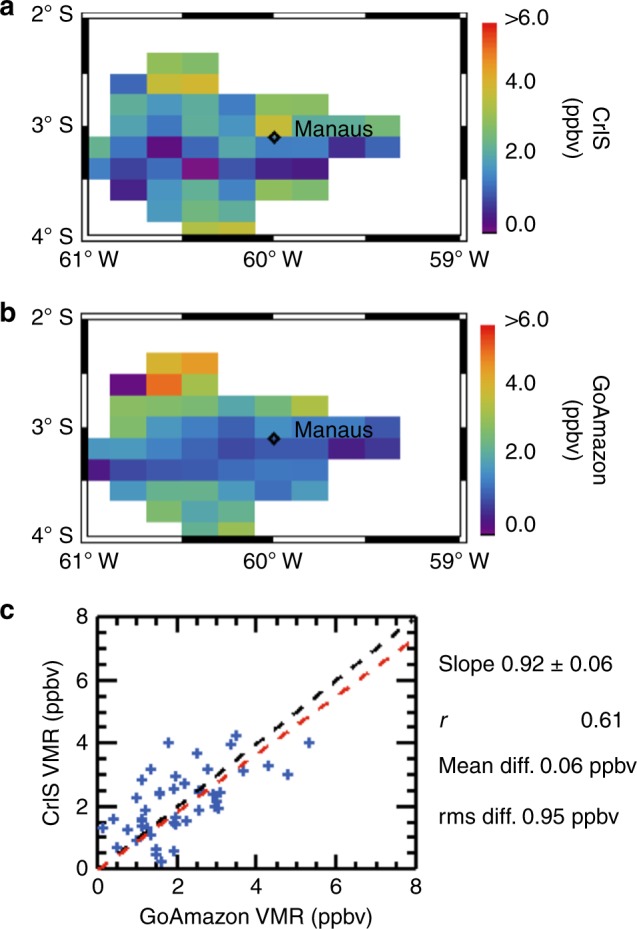
Isoprene concentrations over the Amazonian forest during the GoAmazon flight campaign in September 2014. All data is averaged from the surface to 2 km above ground and mapped on a 0.2° × 0.2° latitude–longitude grid. Plotted are **a** the CrIS isoprene measurements, **b** the GoAmazon airborne proton transfer reaction-mass spectrometry (PTR-MS) measurements, and **c** the CrIS-aircraft correlation. The red dashed line in panel **c** shows the aircraft-satellite linear fit, while the black dashed line indicates the 1:1 relationship

Fig 7a, b show the resulting isoprene distribution as observed by CrIS and measured on-board the aircraft. Because a single, common isoprene concentration profile (blue line in Fig. 4d) was used as a priori (enhanced terrestrial scenario shown in Supplementary Fig. 2b; Supplementary Note 2) for all of the CrIS measurements in this comparison, the retrieved spatial distribution shown in Fig. 7a arises purely from the satellite data. Both CrIS and the GoAmazon aircraft measurements show isoprene enhancements over the northern and southern portions of the survey domain, with lower concentrations in the middle. The CrIS measurements thus capture the elevational isoprene gradient first reported by Gu et al.^34^ over this same region. The satellite:aircraft correlation shows strong overall agreement, with *r* *=* 0.6 (in the range of what is typically found for space-borne observations of formaldehyde^36–38^), slope of 0.92 ± 0.06, and mean and RMS differences of 0.06 and 0.95 ppbv, respectively. In addition to the default 0–2 km vertical filter, we tested other altitude ceilings from 1 to 3 km with an increment of 0.5 km (Supplementary Tables 1 and 2; Supplementary Note 3); both the elevational isoprene gradient and satellite:aircraft comparison are robust across this range.

### Isoprene column sensitivity to OH

**Fig. 8:**
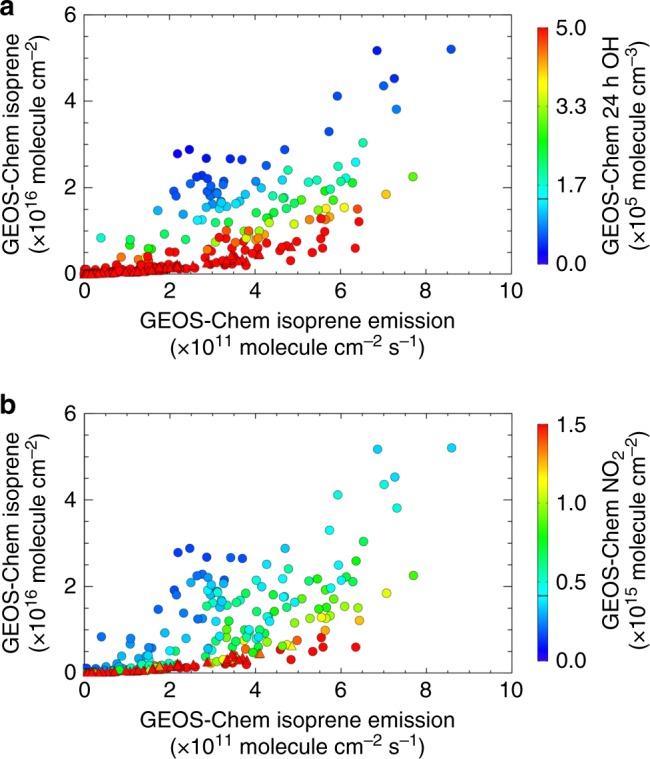
Relationship between isoprene columns and emissions over Amazonia (circles) and the U.S. Southeast (triangles) as simulated by GEOS-Chem. Symbols are colored by **a** model OH (monthly mean below ~500 m) and **b** tropospheric column NO_2_ (monthly mean at the CrIS daytime overpass time)

The strongly enhanced isoprene columns that are both predicted by GEOS-Chem (Figs. 5i and 6c) and observed by CrIS (Fig. 5a) over Amazonia reflect elevated emissions (Fig. 6b), but are also a strong function of the isoprene lifetime (Fig. 6a). Figure 8 quantifies this effect in the model, comparing the isoprene emission:column relationships over Amazonia and the U.S. Southeast. We see that OH in the model is highly suppressed over Amazonia due to strong isoprene emissions coupled with lowatmospheric NO_*x*_ concentrations. The modeled (monthly mean) isoprene lifetime thus exceeds 12 h over much of this region. This yields a positive feedback, in which an emission increase leads to a longer isoprene lifetime, and therefore to a supra-linear concentration increase (Fig. 8). By contrast, predicted isoprene columns are much lower over the U.S. Southeast, despite comparable emission magnitudes—because the modeled isoprene lifetimes are several times shorter than over Amazonia. Here, the predicted isoprene emission:column relationship follows the more linear dependence expected in the absence of a large lifetime feedback (Fig. 8).

The relationship shown in Fig. 8 is a model realization with a treatment of isoprene chemistry reflecting the present state of science (see Methods). However, the degree to which atmospheric OH levels are actually suppressed under high-isoprene, low-NO_*x*_ conditions is an outstanding uncertainty in the community and therefore also in current models^39^. Isoprene’s strong sensitivity to the oxidative regime means that combining space-based isoprene column measurements with contemporaneous observations of its oxidation products can provide a new avenue for understanding this chemistry.

**Fig. 9:**
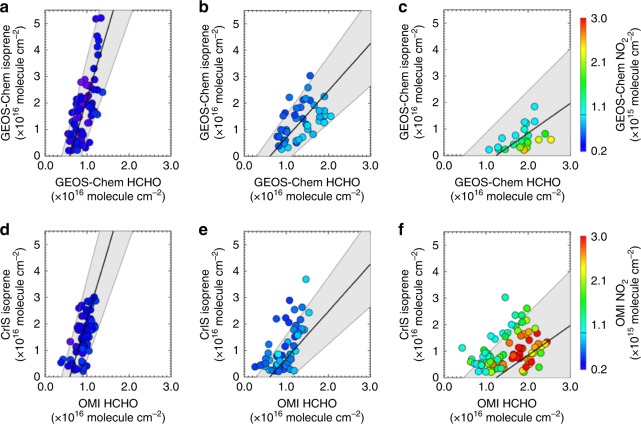
Relationship between isoprene and HCHO as a function of NO_2_ over Amazonia during September 2014. Panels **a**–**c** show the isoprene:HCHO column relationship as simulated by GEOS-Chem over land under conditions of **a** low-NO_2_ column abundance (<0.65 × 10^15^ molecule cm^−2^), **b** moderate NO_2_ (0.65–1.1 × 10^15^ molecule cm^−2^), and **c** high NO_2_ (>1.1 × 10^15^ molecule cm^−2^). The lines and shaded regions indicate the major axis regression and associated 90% confidence interval in each case. Panels **d**–**f** show the measured relationships as observed by the CrIS and OMI satellite sensors superimposed on the corresponding modeled regressions

Figure 9 presents a first demonstration of this potential, showing the isoprene:HCHO relationship as a function of NO_2_, as simulated by GEOS-Chem and as measured by the CrIS and OMI satellite sensors over Amazonia in September 2014 (the OMI HCHO and NO_2_ data are described in Supplementary Note 4). The HCHO columns are less sensitive than isoprene to any potential OH suppression effects, for two reasons. The first is that HCHO production from isoprene is proportional to isoprene × OH, which remains relatively buffered under conditions where isoprene builds up and OH is suppressed. The second reason is that HCHO removal occurs via photolysis as well as via OH, so that daytime concentrations do not tend to build up under low OH since the photolysis sink is still operational.

As a result of these differing sensitivities, the isoprene:HCHO relationship can provide a valuable diagnostic for understanding atmospheric oxidation over isoprene source regions. Panels a–c of Fig. 9 show that the modeled isoprene:HCHO slope shifts to higher values with decreasing NO_2_, reflecting suppressed OH and longer isoprene lifetimes when isoprene concentrations are high and NO_*x*_ is low. Panels d–f of Fig. 9 show the corresponding isoprene:HCHO regimes measured from space by CrIS and OMI, overlaid on the modeled regression fit in each case. Under the lowest-NO_*x*_ conditions, the isoprene:HCHO relationship is consistent with that simulated by the model, thus supporting the degree of OH suppression/recycling in the chemical mechanism that is shown in Fig. 8. The observations show slightly more scatter above the modeled isoprene:HCHO slope under higher-NO_2_ conditions. However, in none of the regimes do the observations point to a lower isoprene:HCHO slope and thus to a greater degree of OH recycling than is currently present in the model.

## Discussion

This paper reports the first direct measurements of isoprene from space, using CrIS—a nadir-viewing thermal IR imaging spectrometer. We present a full-physics algorithm that can precisely and accurately retrieve atmospheric isoprene abundances while properly accounting for the relevant spectral interferences. We find that the CrIS retrievals are primarily sensitive to isoprene in the 825–990 hPa range, and have DOF for signal approaching 1.0 over portions of Amazonia where isoprene is high. A comparison between the isoprene column amounts measured by CrIS and those predicted by GEOS-Chem over Amazonia (in September 2014) reveals some coherence (*r* *=* 0.77, slope = 0.87 ± 0.03), but also some important spatial biases in the model that call for further investigation. Aircraft measurements of isoprene conducted during the GoAmazon campaign show strong agreement with the CrIS retrievals (slope = 0.92 ± 0.06, RMS difference < 1 ppbv), supporting the utility and reliability of the CrIS isoprene data. Finally, a first application combining space-based isoprene and formaldehyde measurements to constrain atmospheric oxidation shows broad agreement with model predictions under high-isoprene, low-NO_*x*_ conditions.

The work reported here lays the groundwork for future studies applying the CrIS isoprene data to improve scientific understanding of isoprene emissions and of the atmospheric chemistry that ensues. Suomi-NPP is the predecessor to a series of next-generation U.S. weather satellites in the Joint Polar Satellite System (JPSS). The methodology presented in this work can be applied to measurements from the second CrIS instrument onboard the JPSS-1 platform (launched in November 2017), and from the third CrIS instrument onboard JPSS-2 (scheduled to launch in 2022). Combining these measurements could then provide global isoprene measurements spanning multiple decades.

## Methods

### Radiative transfer modeling

We employ the Earth Limb and Nadir Operational Retrieval (ELANOR)^40–42^ RTM for the observing system simulation experiment (OSSE) and for the MUSES retrieval algorithm^21–24^. ELANOR enables full-physics radiative transfer modeling in the thermal IR spectral region, including upwelling atmospheric emission, down-welling and back-reflected atmospheric emission, surface emission^40^, and cloud properties^41,42^. The contribution from clouds is parameterized in the RTM via a set of frequency-dependent nonscattering optical depths and a cloud top pressure. This is necessary because the initial brightness temperature-based cloud screening described in the main text, while providing a first-order cloud filter, can miss thin clouds and does not provide the frequency-dependent cloud spectra needed to account quantitatively for the resulting impacts on isoprene retrievals. As part of the MUSES algorithm, ELANOR operationally provides simulated spectral radiances and associated Jacobians with respect to targeted parameters to enable data production from a suite of space-borne instruments^21–24^.

In this study, the radiative transfer calculation uses a 66-layer grid at fixed pressure levels. The pressure at the Earth’s surface provides the lower boundary for the forward model and is defined for every GEOS-Chem grid cell (for the OSSE) or individual CrIS observation (for the isoprene retrievals). Sea-level pressures are obtained from version 5 of the NASA Global Modeling and Assimilation Office (GMAO) Goddard Earth Observing System (GEOS-5) model^43^. Surface pressures are then calculated from the geodetic elevation of each location based on these sea-level pressures and the hydrostatic equation. The global MODIS-derived emissivity database from the University of Wisconsin, Madison^44^ is used to provide spectrally varying IR surface emissivities. Atmospheric temperature profiles are from GEOS-5^43^. Nonisoprene trace gas profiles employ an offline climatology from the Model for OZone and Related chemical Tracers (MOZART)-4^45^ (those for isoprene are as described in Supplementary Note 2). The above emissivities, temperatures, and trace gas profiles are used as input for the OSSE and as a priori for the MUSES retrievals. Precalculated look-up tables of molecular absorption are used in the simulation of radiances and weighting functions in the spectral regions of interest (Table 1 in the main text). These tables are calculated using the line by line radiative transfer model (LBLRTM)^46^. Spectroscopic parameters for isoprene are from Brauer et al.^15^, and those for nonisoprene species relevant to this work are from the AER v3.4 line parameter database (http://rtweb.aer.com), which is based on the HIgh-resolution TRANsmission molecular absorption (HITRAN) 2012 database^47^.

### MUSES algorithm

The MUSES isoprene algorithm is a full-physics retrieval developed for this work. The MUSES framework has been applied previously to accommodate multiple instruments in quantifying the vertical distribution of other tropospheric species, including joint joint CrIS + TROPOMI CO profiling^21^, AIRS + OMI O_3_ retrievals^22^, and TES + OMI O_3_ retrievals^23^, and to estimate the deuterium content of atmospheric water vapor^24^. The development of MUSES leverages a suite of forward RTMs, including ELANOR^42,43,46^ for simulating thermal IR radiances and Jacobians^21–24^; the U.S. Smithsonian Astrophysical Observatory OMI OZone PROFile (PROFOZ) algorithm^48^ for simulating UV radiances and Jacobians in the Hartley and Huggins bands^22,23,49^; and the full-physics OCO-2 algorithm^50,51^ for simulating short-wavelength IR radiances and Jacobians^21^.

The MUSES retrieval approach is based on the optimal estimation method^33^, which minimizes the differences between observed and simulated radiances subject to a priori knowledge (i.e., the mean and covariance of the atmospheric-cloud-surface state) to infer the optimal or maximum a posteriori solution. The retrieval equations of relevance for CrIS measurements are detailed by Fu et al.^21^. The use of optimal estimation in the MUSES algorithm also enables computation of the averaging kernel and error matrices for each individual sounding—which is important information needed for trend analysis, atmospheric model evaluation, and data assimilation. Equations used in MUSES to compute the averaging kernel matrix (**A**) and total error covariance matrix (**S**) are provided by Fu et al.^21,22^. We note that while the MUSES retrieval scheme does not include RTM error, this is expected to be negligible relative to other error sources given the demonstrated accuracy of the ELANOR full-physics RTM in the thermal IR^40,46^.

### GEOS-Chem chemical transport model

GEOS-Chem (v11-02e; www.geos-chem.org) is a 3D global Eulerian CTM, here driven by GEOS-FP assimilated meteorological data from NASA GMAO. The GEOS-FP have native horizontal resolution of 0.25° × 0.3125° (latitude × longitude) with 72 vertical levels, 1-hourly temporal resolution for surface variables and mixing depths, and 3-hourly temporal resolution for 3D meteorological parameters. The global simulations presented here employ a horizontal resolution of 2.0° × 2.5° with 47 vertical levels (~14 are below 2 km altitude) and a 10–15 min transport timestep. The model uses the TPCORE advection algorithm^52^, convective mass fluxes from the GEOS-FP archive^53^, nonlocal boundary layer mixing as described by Lin and McElroy^54^, and wet and dry deposition as described by Amos et al.^55^ and Wang et al.^56^. A 1-year spin-up is used for initialization.

The model includes comprehensive HO_*x*_-NO_*x*_-VOC-O_3_ chemistry coupled to aerosols^31–33^, and follows current JPL/IUPAC recommendations. The chemical mechanism includes extensive recent updates related to isoprene^30–32,57,58^ based on recent laboratory and field-based findings. These include updated isoprene hydroxyhydroperoxide (ISOPOOH) yields from the reaction of isoprene peroxy radicals (ISOPO_2_) with HO_2_^59^; updated rates and products for the reaction of isoprene epoxides (IEPOX) with OH^60^; and further updates related to ISOPO_2_ self-reaction^61^, aerosol uptake of isoprene oxidation products^62^, and isoprene nitrate chemistry^32^. Isomerization of ISOPO_2_ is treated explicitly, with oxidation and photolysis of the resulting hydroperoxyaldehydes following the current state-of-science^63–68^ as described by Fisher et al.^32^. The isoprene mechanism employed here (GEOS-Chem v11-02e) is consistent with that in the GEOS-Chem (version v11-02c) mechanism evaluated in detail by Bates and Jacob^69^. We refer the reader to that publication for more details on the associated OH suppression/recycling and other chemical impacts of isoprene oxidation.

Biogenic emissions of isoprene and other VOCs are simulated using MEGANv2.1^1^, implemented in GEOS-Chem as described by Hu et al.^70^. Global anthropogenic emissions are based on the RETRO inventory for VOCs and on EDGARv4.2 (http://edgar.jrc.ec.europa.eu) for NO_*x*_, SO_*x*_, and CO; each is overwritten by regional inventories where appropriate. Global Fire Emission Database (GFED)^71^ is used to compute biomass burning emissions.

## Supplementary information

Supplementary Information

## Data Availability

CrIS L1B data used in this work is publicly available at https://disc.gsfc.nasa.gov/datacollection/SNPPCrISL1BNSR_1.html, Accessed: August 6, 2019. The data were produced by Dr. Hank Revercomb at UW-Madison Space Science and Engineering Center; and Dr. Larrabee Strow at UMBC Atmospheric Spectroscopy Laboratory. All of the airborne measurement data used here is available at http://www.arm.gov/campaigns/amf2014goamazon*.* Other data supporting the findings of this study are available from the authors on reasonable request; see author contributions for specific data sets.

## Code availability

The LBLRTM^41,47^, which provides the radiative transfer modeling capabilities of the ELANOR model^41–43^ used in this work, is publicly available at http://rtweb.aer.com/lblrtm.html. The MUSES algorithm will be publicly accessible pending required U.S. governmental review. GEOS-Chem model code is publicly available at www.geos-chem.org.
