# Supplementary material for: Direct retrieval of isoprene from satellite-based infrared measurements
Source: Nat Commun. 2019 Aug 23;10:3811. doi: 10.1038/s41467-019-11835-0 (PMC6707292; doi:10.1038/s41467-019-11835-0)
Supplement: Supplementary file 1 — Supplementary Information [file 41467_2019_11835_MOESM1_ESM.pdf]

Cover Page

Direct retrieval of isoprene from satellite-based infrared measurements

Fu et al.

Supplementary Note 1: Potential for other VOCs to interfere with the isoprene spectral signal.

We investigated the potential for other VOCs to interfere with the isoprene signal used here. Compounds that are also emitted from vegetation, that contain carbon double bond structures analogous to those causing isoprene's ν_{27} and ν_{28} features (terminal $=CH_2$ moieties), and that have published absorption cross-sections in the relevant spectral region include ethene, β-pinene, d-limonene, and myrcene. Supplementary Figure 1 shows that, based on the HITRAN 2016 database[72], the absorption cross-sections for β-pinene, d-limonene, and myrcene (and also α-pinene) are approximately 4-100 times smaller than that of isoprene across the spectral region of interest. Furthermore, expected concentrations of these species over Amazonia are significantly lower than that of isoprene: for example, Yáñez-Serrano et al.[73] report measured concentrations at the Amazon Tall Tower Observatory that are typically on the order of tenths of ppb for each of these species. The resulting spectral signal is approximately one order of magnitude smaller than that of isoprene, and hence not of major importance for the location and timeframe of our study. In the case of ethene, the major spectral feature (which has been exploited for space-based[74-75] and ground-based[76] remote sensing measurements) resides at 949.3 – 949.5 cm^{-1}, outside the relevant spectral window for our study. Additional biogenic VOCs with terminal $=CH_2$ groups include propene, sabinene, and t-β-ocimene; thermal infrared absorption cross sections for these species are not currently available. Such laboratory measurements should be prioritized in the future, but based on our findings for ethene, α/β-pinene, d-limonene, and myrcene we expect their impact to likewise be minor over our study domain.

Supplementary Note 2: A priori profiles and constraints.

Atmospheric isoprene is known to vary substantially both geographically and seasonally[77-80]. Accordingly, the GEOS-Chem simulation described in the Methods section is used here to generate a priori profiles and constraint matrices for the CrIS isoprene retrieval. Three a priori profiles (representing enhanced terrestrial, moderate terrestrial, and clean conditions) spanning both land and ocean scenes were generated from GEOS-Chem model output on a 2° × 2.5° latitude-longitude grid for 2013. The enhanced terrestrial profiles correspond to scenarios with isoprene concentrations exceeding 3.0 ppbv. Profiles over land or ocean were classified as clean if the surface concentration was below 0.1 ppbv. Terrestrial scenes meeting neither of these criteria were classified as moderate. Supplementary Figure 2 shows the individual and mean profiles for each category.

We employ these mean profiles as a priori in the CrIS isoprene retrieval, with each model grid box and month assigned one of the three. At the start of the CrIS retrieval, the observation coordinates and month are used to select the appropriate stored a priori profile; this initiates the retrieval in the most likely region in retrieval space, and increases the probability of finding an optimal solution. The initial guess for each retrieval is always set to an enhanced profile in order to avoid falling into null space on the first retrieval step. Note that the a priori profile may thus differ from the initial guess. The variability in each of the three a priori profiles is set to 100% for pressure levels between 0 and 2 km above the surface. Since the CrIS retrieval algorithms operate in log space to accommodate the wide range of atmospheric isoprene concentrations and to avoid negative results, the

constraint matrices and the averaging kernels are also calculated in this manner. The diagonals are modified to reflect the CrIS sensitivity: where isoprene is generally negligible (e.g., >4 km above ground), the variability is reduced to 0.1% to obtain tighter constraints at levels with limited information from CrIS. This reduction was achieved by applying a Gaussian-based tapering to the diagonals of the covariance matrix. Off-diagonal elements of the constraint matrix were generated based on a 2-km correlation length.

Supplementary Note 3: Vertical distribution of isoprene measured by airborne PTR-MS instrument and the impacts of altitude range on the satellite:aircraft correlation.

Supplementary Figure 3 shows that, based on the airborne PTR-MS measurements[81], the majority of the isoprene burden resides in the surface – 2 km altitude range. Supplementary Figure 4 shows the spatial coverage of airborne PTR-MS measurements as a function of altitude; measurements above 2 km are sparser than at lower altitudes. Supplementary Figure 5 and Supplementary Table 1 show that the spatial distribution of isoprene concentrations as measured by airborne PTR-MS is robust across the choice of altitude ranges from 0 – 1 km to 0 – 3 km.

Supplementary Table 2 shows that the satellite-aircraft comparison does not differ strongly across these altitude ranges, though the correlation does decrease somewhat when the vertical sampling is restricted to 0 –1 km.

Supplementary Note 4: HCHO and NO_2 column observations from the Ozone Monitoring Instrument (OMI).

OMI[82-84] is a nadir-viewing imaging spectrometer on board the NASA Aura satellite, with a sun-synchronous polar orbit and equator crossing time of 13:42 LT (ascending node). OMI measures UV/Visible solar back-scattered radiances from 270 to 500 nm in three spectral channels. The instrument has 115° cross-track field of view, which results in a 2,600 km ground swath of 60 pixels (from 13×24 km^2 at nadir to 26×135 km^2 at swath edges) that provides daily global coverage.

For both HCHO and NO_2 we use data products provided by the Belgian Institute for Space Aeronomy, BIRA (v14 for HCHO[84], QA4ECV for NO_2[85]; the latter is a joint product of BIRA, KNMI, the University of Bremen, the Max Planck Institute for Chemistry, and Wageningen University). The monthly mean data have been subjected to the recommended screening criteria (for solar zenith angle, cloud fraction, quality flags, row anomaly removal) in each case, and we grid them here to the GEOS-Chem model resolution. For HCHO we follow standard procedures for reference sector correction[86-87] and apply the 20% bias correction recommended by Zhu et al.[88] for this product.

Supplementary References

72. Gordon, I.E. et al. The HITRAN 2016 molecular spectroscopic database. *JQSRT* **203**, 3-69 (2017).

73. Yáñez-Serrano, A.M. et al. Monoterpene chemical speciation in a tropical rainforest: variation with season, height, and time of day at the Amazon Tall Tower Observatory (ATTO). *Atmos. Chem. Phys.* **18**, 3403-3418 (2018).

74. Coheur, P.-F. et al. IASI measurements of reactive trace species in biomass burning plumes. *Atmos. Chem. Phys.* **9**, 5655–5667 (2009).

75. Dolan, W. et al. Satellite observations of ethylene (C_2H_4) from the Aura Tropospheric Emission Spectrometer: A scoping study. *Atmos. Env.* **141**, 388-393 (2016).

76. Toon, G.C. et al. Measurements of atmospheric ethene by solar absorption FTIR spectrometry. *Atmos. Chem. Phys.* **18**, 5075–5088 (2018).

77. Palmer, P.I. et al. Quantifying the seasonal and interannual variability of North American isoprene emissions using satellite observations of the formaldehyde column. *J. Geophys. Res.* **111,** D12315 (2006).

78. Millet, D.B. et al., Spatial distribution of isoprene emissions from North America derived from formaldehyde column measurements by the OMI satellite sensor. *J. Geophys. Res.* **113,** D02307 (2008).

79. Barkley, M.P. et al. Top-down isoprene emissions over tropical South America inferred from SCIAMACHY and OMI formaldehyde columns. *J. Geophys. Res.* **118,** 6849–6868 (2013).

80. Stavrakou, T. et al. Isoprene emissions over Asia 1979–2012: impact of climate and land-use changes. *Atmos. Chem. Phys.* **14,** 4587–4605 (2014).

81. Shilling, J.E. et al. Aircraft observations of the chemical composition and aging of aerosol in the Manaus urban plume during GoAmazon 2014/5. *Atmos. Chem. Phys.* **18,** 10773-10797 (2018).

82. Levelt, P. F. et al. The Ozone Monitoring Instrument. *IEEE Trans. Geosci. Remote* **44**, 1093–1101 (2006).

83. Levelt, P.F., et al. The Ozone Monitoring Instrument: overview of 14 years in space. *Atmos. Chem. Phys.* **18,** 5699-5745 (2018).

84. De Smedt, I., et al. Improved retrieval of global tropospheric formaldehyde columns from GOME-2/MetOp-A addressing noise reduction and instrumental degradation issues. *Atmos. Meas. Tech.*, **5**, 2933-2949 (2012).

85. Boersma, K. F. et al. QA4ECV NO_2 tropospheric and stratospheric vertical column data from OMI (Version 1.1). Royal Netherlands Meteorological Institute (KNMI). (2017).

86. Marais, E.A. et al. Isoprene emissions in Africa inferred from OMI observations of formaldehyde columns. *Atmos. Chem. Phys.* **12**, 6219-6235 (2012).

87. Shen, L. et al. The 2005–2016 trends of formaldehyde columns over China observed by satellites: Increasing anthropogenic emissions of volatile organic compounds and decreasing agricultural fire emissions. *Geo. Res. Lett.* **46**, 4468–4475 (2019).

88. Zhu, L. et al. Observing atmospheric formaldehyde (HCHO) from space: validation and intercomparison of six retrievals from four satellites (OMI, GOME2A, GOME2B, OMPS) with SEAC4RS aircraft observations over the southeast US, *Atmos. Chem. Phys.* **16**, 13477-13490 (2016).

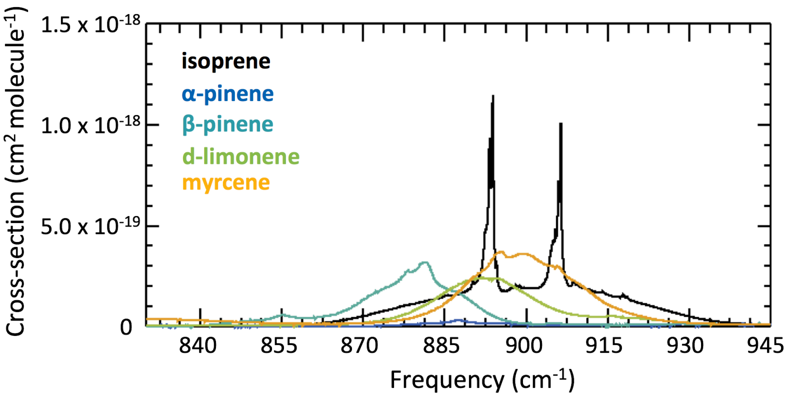

Supplementary Figure 1 | Absorption cross-sections of isoprene, α-pinene, β-pinene, d-limonene, and myrcene. Data plotted is from the HITRAN 2016 database (https://hitran.org/xsc/, accessed: June 3, 2019).

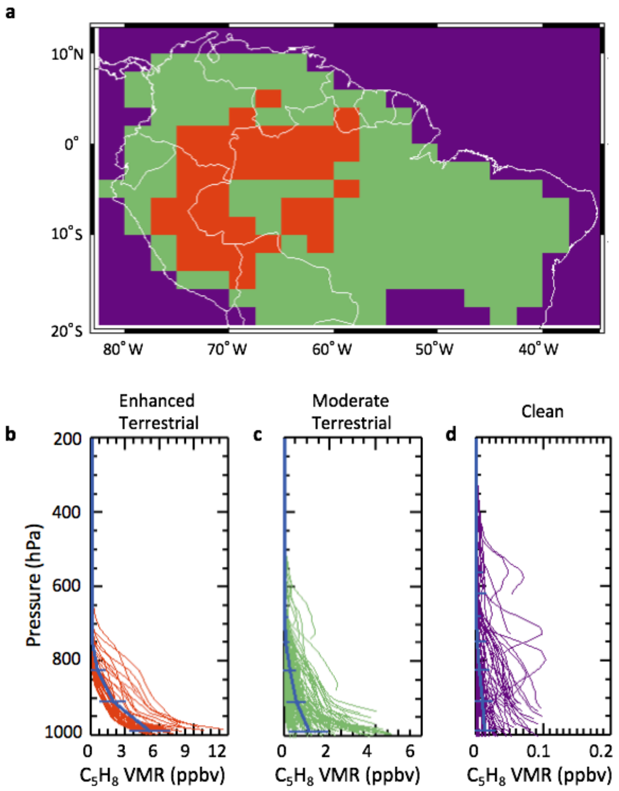

Supplementary Figure 2 | Isoprene volume mixing ratio (VMR) profiles predicted by GEOS-Chem over Amazonia for 2013. (a) spatial distribution binned by type (red: enhanced terrestrial, green: moderate terrestrial, purple: clean land and ocean). **(b)** enhanced terrestrial**. (c)** moderate terrestrial**. (d)** clean land and ocean. The mean profiles for each category, shown in blue, are used as a priori profiles in the CrIS isoprene retrievals. Blue horizonal bars indicate the standard deviation for each level. The enhanced terrestrial, moderate terrestrial, and clean scenarios represent 13.5%, 41.2%, and 45.3%, respectively, of the model profiles for the region shown.

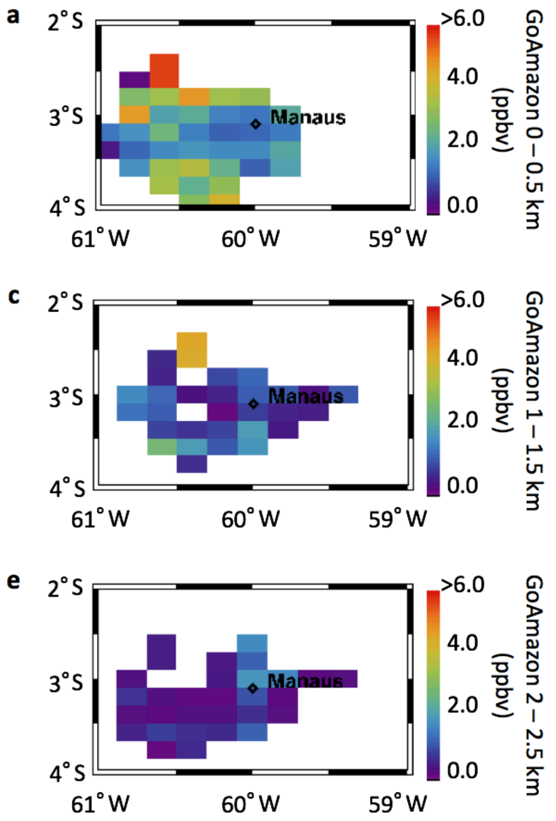

Supplementary Figure 3 | Isoprene concentrations measured by airborne PTR-MS during the GoAmazon campaign in September 2014. All data is on a 0.2º × 0.2º latitude-longitude grid. Plotted are the airborne PTR-MS measurements in six altitude ranges: **(a)** surface to 0.5 km, **(b)** 0.5 km to 1.0 km, (**c**) 1.0 to 1.5 km, **(d)** 1.5 km to 2.0 km, (**e**) 2.0 to 2.5 km, **(f)** 2.5 km to 3.0 km.

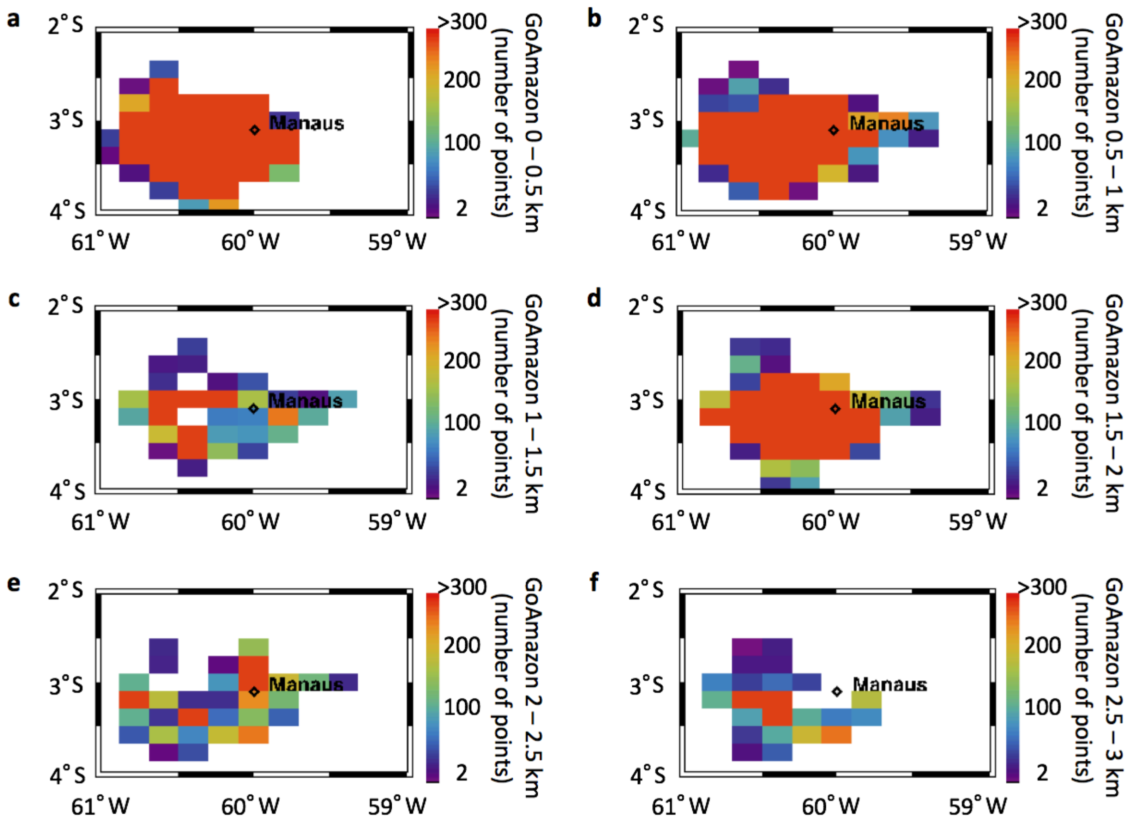

Supplementary Figure 4 | Spatial coverage of airborne PTR-MS measurements during the GoAmazon campaign in September 2014. All data is mapped on a 0.2º × 0.2º latitude-longitude grid. Plotted is the number of data points in six altitude ranges: **(a)** surface to 0.5 km, **(b)** 0.5 km to 1.0 km, (**c)** 1.0 to 1.5 km, **(d)** 1.5 km to 2.0 km, (**e)** 2.0 to 2.5 km, **(f)** 2.5 km to 3.0 km.

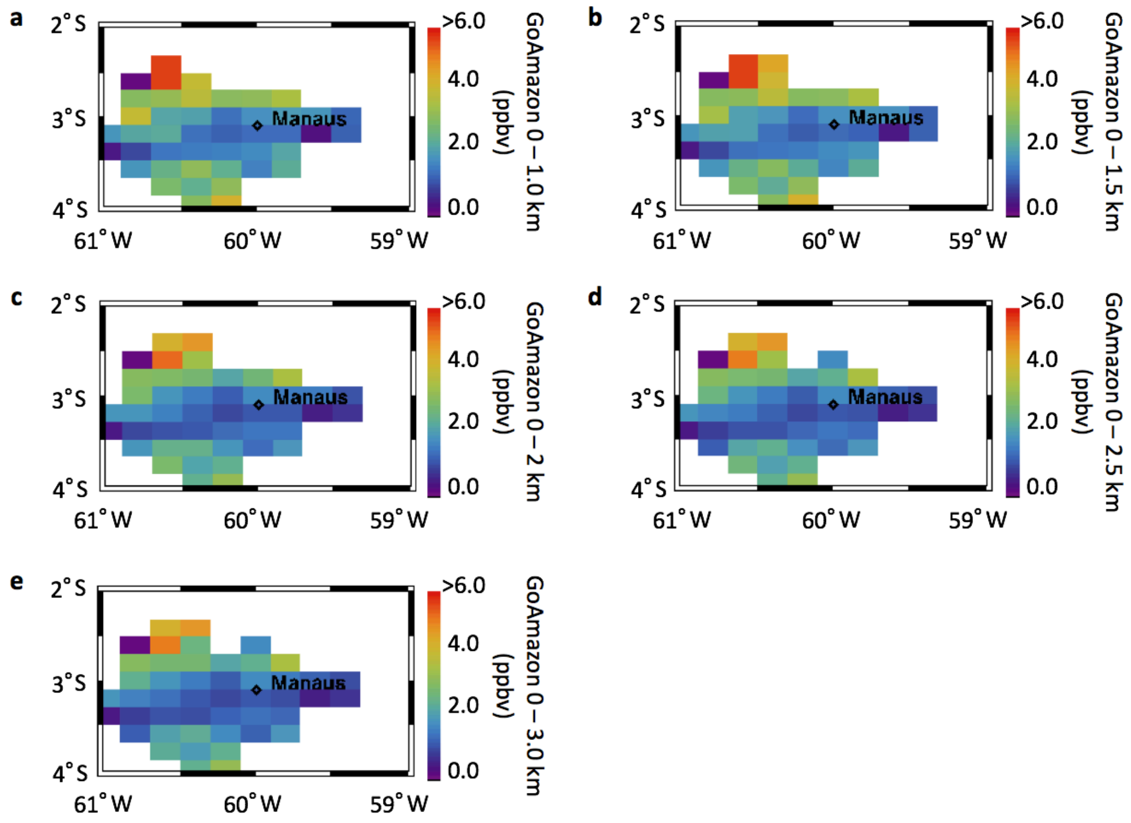

Supplementary Figure 5 | Isoprene concentrations measured by airborne PTR-MS during the GoAmazon campaign in September 2014. All data is on a 0.2º × 0.2º latitude-longitude grid. Plotted are the GoAmazon airborne proton transfer reaction-mass spectrometry (PTR-MS) measurements in five altitude ranges: (**a**) surface to 1.0 km, **(b)** surface to 1.5 km, (**c**) surface to 2.0 km, **(d)** surface to 2.5 km, (**e**) surface to 3.0 km.

Supplementary Table 1 | Consistency of the horizontal isoprene distribution as a function of altitude based on airborne PTR-MS measurements during the GoAmazon campaign in September 2014 [a]

Altitude range (km)	0 - 1.0	0 - 1.5	0 - 2.5	0 - 3.0
Slope	1.19 ± 0.02	1.15 ± 0.02	0.97 ± 0.01	0.94 ± 0.01
r	0.77	0.97	0.99	0.99
Mean diff. (ppbv)	-0.29	-0.33	0.07	0.12
RMS diff. (ppbv)	0.85	0.34	0.14	0.19

[a] Values shown represent a comparison of the 0 - 2 km distribution with those for the four other altitude ranges indicated in the table.

Supplementary Table 2 | Consistency of the CrIS-aircraft isoprene comparison as a function of altitude [a]

Altitude range (km)	0 - 1.0	0 - 1.5	0 - 2.0	0 - 2.5	0 - 3.0
Slope	0.79 ± 0.05	0.80 ± 0.05	0.92 ± 0.06	0.95 ± 0.06	0.98 ± 0.06
r	0.52	0.61	0.61	0.64	0.63
Mean diff. (ppbv)	-0.22	-0.27	-0.27	0.13	0.19
RMS diff. (ppbv)	1.18	1.05	1.05	0.92	0.92

[a] Values shown represent regression statistics between airborne PTR-MS and spaceborne CrIS measurements during the GoAmazon campaign in September 2014 for the indicated altitude ranges.
